# Organizing pneumonia after thoracic radiotherapy followed by anti‐PD‐1 antibody treatment for patients with lung cancer: Three case reports

**DOI:** 10.1111/1759-7714.13102

**Published:** 2019-05-23

**Authors:** Masakuni Sakaguchi, Toshiya Maebayashi, Takuya Aizawa, Naoya Ishibashi, Masahiro Okada

**Affiliations:** ^1^ Department of Radiology Nihon University School of Medicine Tokyo Japan

**Keywords:** Anti‐PD‐1 antibody, organizing pneumonia (OP), programmed cell death protein 1 (PD‐1), thoracic radiotherapy (TRT)

## Abstract

Anti‐PD‐1 antibodies and thoracic radiation therapy (TRT) generate adverse events, including pneumonitis. However, there is limited information about potential overlapping toxicity of anti‐PD‐1 antibodies administered after TRT. Herein, we report three cases. The first case was of a man in his 80s with squamous cell lung cancer (cT2aN0M0 stage IB). Twelve months after TRT, tumor regrowth was observed, and the patient was administered nivolumab. Twenty‐four months after TRT, computed tomography (CT) showed organizing pneumonia (OP). The second case was of a man in his 70s with squamous cell lung cancer. He underwent surgery for pT3N1M0 stage IIIA; however, mediastinum lymph node metastasis developed. Therefore, he received TRT for the mediastinum lymph node metastasis. One month after the completion of TRT, nivolumab was administered. Two months after TRT, an OP diagnosis was made. The third case was of a man in his 60s with an unknown type of lung cancer. He received TRT for cT4N2M0 stage IIIB. Fourteen months after TRT, tumor regrowth was observed, thus, nivolumab was administered. Twenty‐seven months after TRT, an OP diagnosis was made. These case reports draw attention to OP after TRT and anti‐PD‐1 antibody administration despite low V20. Careful follow‐up of such patients is advised considering synergistic adverse events.

## Introduction

Pembrolizumab and nivolumab are immune checkpoint monoclonal antibodies that bind the PD‐1 receptor, thereby blocking the binding of PD‐1 and its ligands PD‐L1/PD‐L2.^1^, ^2^, ^3^, ^4^ Despite impressive clinical benefits, these immune checkpoint inhibitors can cause many immune‐related adverse events (irAEs).^5^ A previous report demonstrated that the incidence of any grade pneumonitis associated with PD‐1 inhibitor monotherapy in patients with NSCLC was 4.1% (1.4–8.5%).^6^ In patients with NSCLC, lung damage resulting from thoracic radiotherapy (TRT) prior to anti‐PD‐1 antibody administration could affect the pneumonitis incidence rate. However, there is limited information about potential overlapping toxicity of anti‐PD‐1 antibodies administered after TRT. Herein we report the cases of three patients who developed pneumonitis after anti‐PD‐1 antibody treatment following TRT.

### Case presentation

During April 2014 and June 2018, 241 patients received TRT for lung cancer at our institute and 27 (11.2%) developed grade ≥ 2 pneumonitis according to the Common Terminology Criteria for Adverse Events (CTCAE) version 4.0.^7^ Furthermore, 15 patients received anti‐PD‐1 antibody treatment after TRT, of which three (20%) developed grade ≥ 2 organizing pneumonia (OP). All three patients had pneumonitis inside, outside, or both inside and outside of the irradiation field, which had migrated to the opposite lung, leading to an OP diagnosis. All patients had a smoking history but the lung volume covered by ≥ 20 Gy (V20) was reduced to a safe range and respiratory function was almost within normal limits considering their age.

#### Case 1

**Figure 1:**
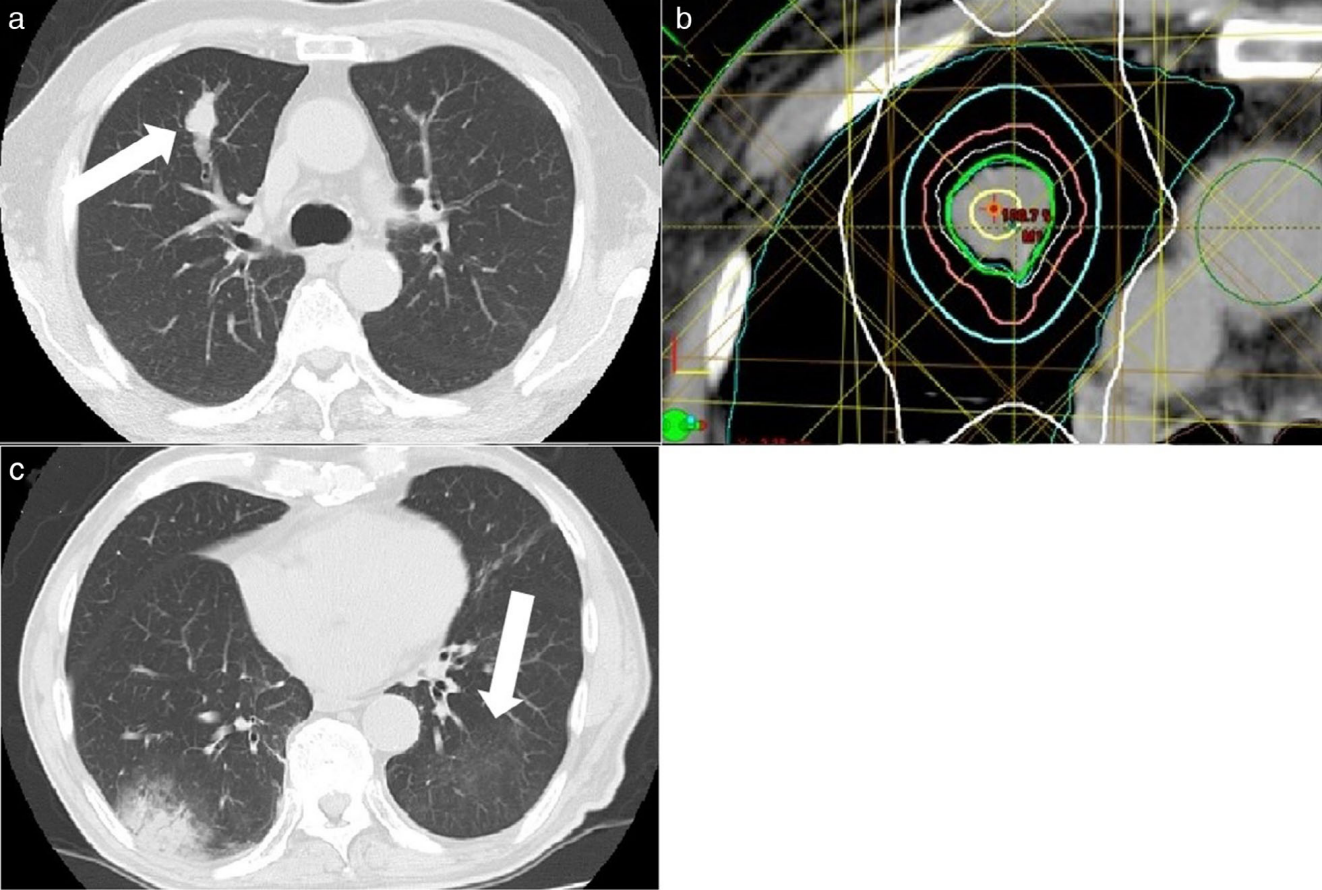
(**a**) Computed tomography (CT) shows primary lung cancer in the right upper lobe (arrow) in a male patient in his 80s. (**b**) He received three‐dimensional conformal radiotherapy; isodose lines of 100% (yellow), 95% (light green), 85% (light blue), 50% (white), and the planned target volume (pink) are shown. (**c**) After 13 courses of an anti‐PD‐1 antibody (24 months after thoracic radiation therapy), CT showed newly developed patchy opacity with air bronchograms in the irradiated area, which had spread to the low‐dose area and outside the irradiated area. Migration of lung infiltration with ground‐glass opacity in the left lung (arrow) was observed and diagnosed as organizing pneumonitis.

The first case was of a man in his 80s with squamous cell lung cancer of the right upper lobe (Fig 1a). The tumor was cT2aN0M0 stage IB according to the Union for International Cancer Control (UICC) 7th edition. We decided to administer TRT at a total dose of 60 Gy in 12 fractions (Fig 1b). Twelve months after TRT, tumor regrowth was observed, and the patient was administered nivolumab at a dose of 3 mg/kg (170 mg). After 13 courses of nivolumab (24 months after TRT), the patient experienced discomfort in the anterior chest. A diagnosis of grade 3 OP was made (Fig 1c).

#### Case 2

**Figure 2:**
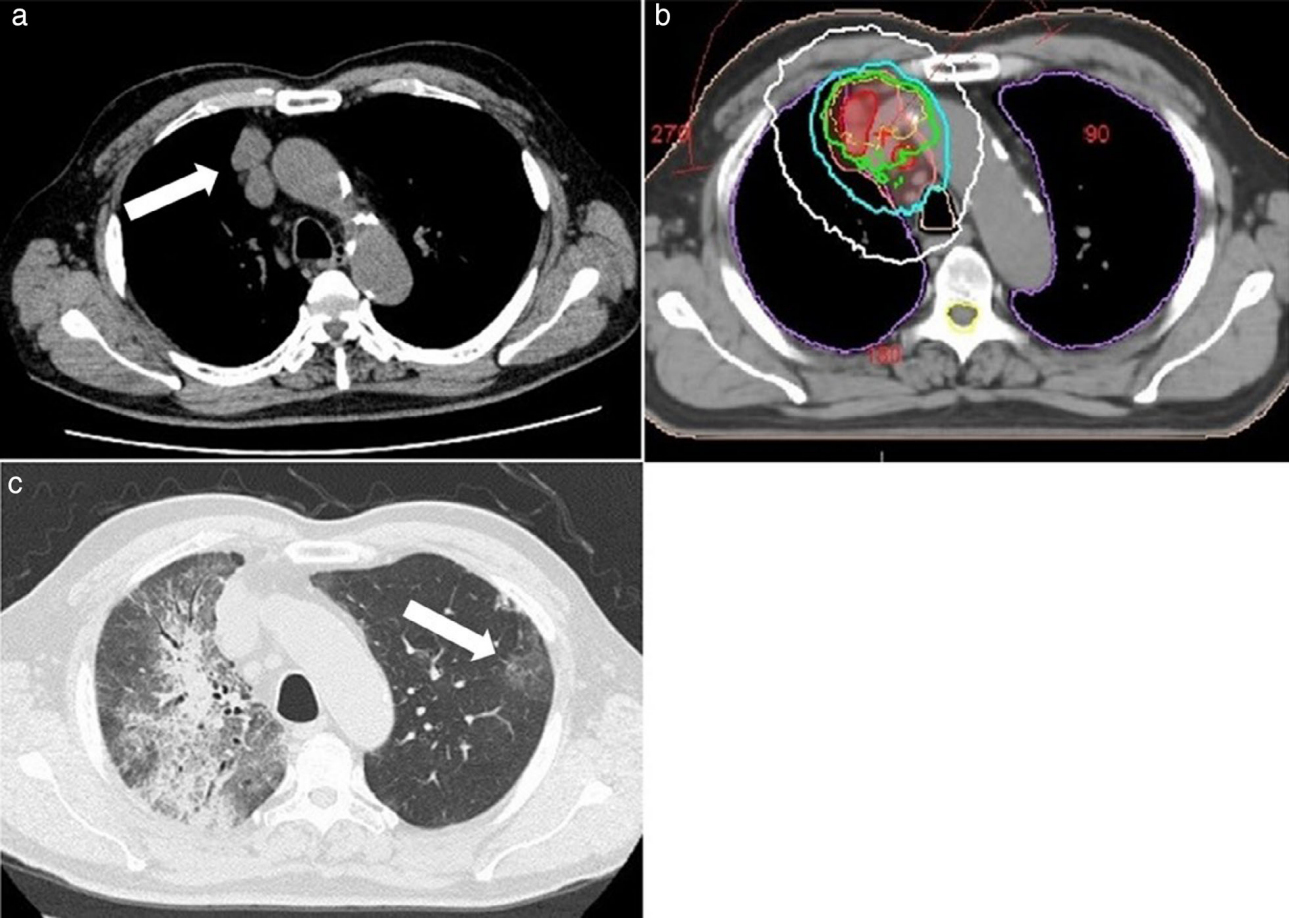
(**a**) Computed tomography (CT) shows the recurrence of mediastinum lymph node metastases (arrow) in a man in his 70s. (**b**) He received three‐dimensional radiotherapy; isodose lines of 100% (yellow), 95% (light green), 85% (light blue), 50% (white), and the planned target volume (pink) are shown. (**c**) After one course of an anti‐PD‐1 antibody (2 months after thoracic radiation therapy), CT showed newly developed patchy opacity with air bronchograms in the irradiated area, which had also spread to the low‐dose area and outside the irradiated area. Ground‐glass opacity of the left lung (arrow) was also observed and diagnosed as organizing pneumonitis.

The second case was of a man in his 70s with squamous cell lung cancer of the right lower lobe. He underwent surgery for pT3N1M0 stage IIIA NSCLC. However, mediastinum lymph node metastasis developed near the surgical area after neoadjuvant chemotherapy (Fig 2a). He was administered TRT at a total dose of 45 Gy in 15 fractions (Fig 2b). One month after the completion of three‐dimensional conformal radiotherapy (3D‐CRT), nivolumab was administered at 3 mg/kg (240 mg). After the first nivolumab treatment (2 months after 3D‐CRT), the patient presented with a dry cough and dyspnea. A clinical diagnosis of grade 3 OP was made (Fig 2c).^7^

#### Case 3

**Figure 3:**
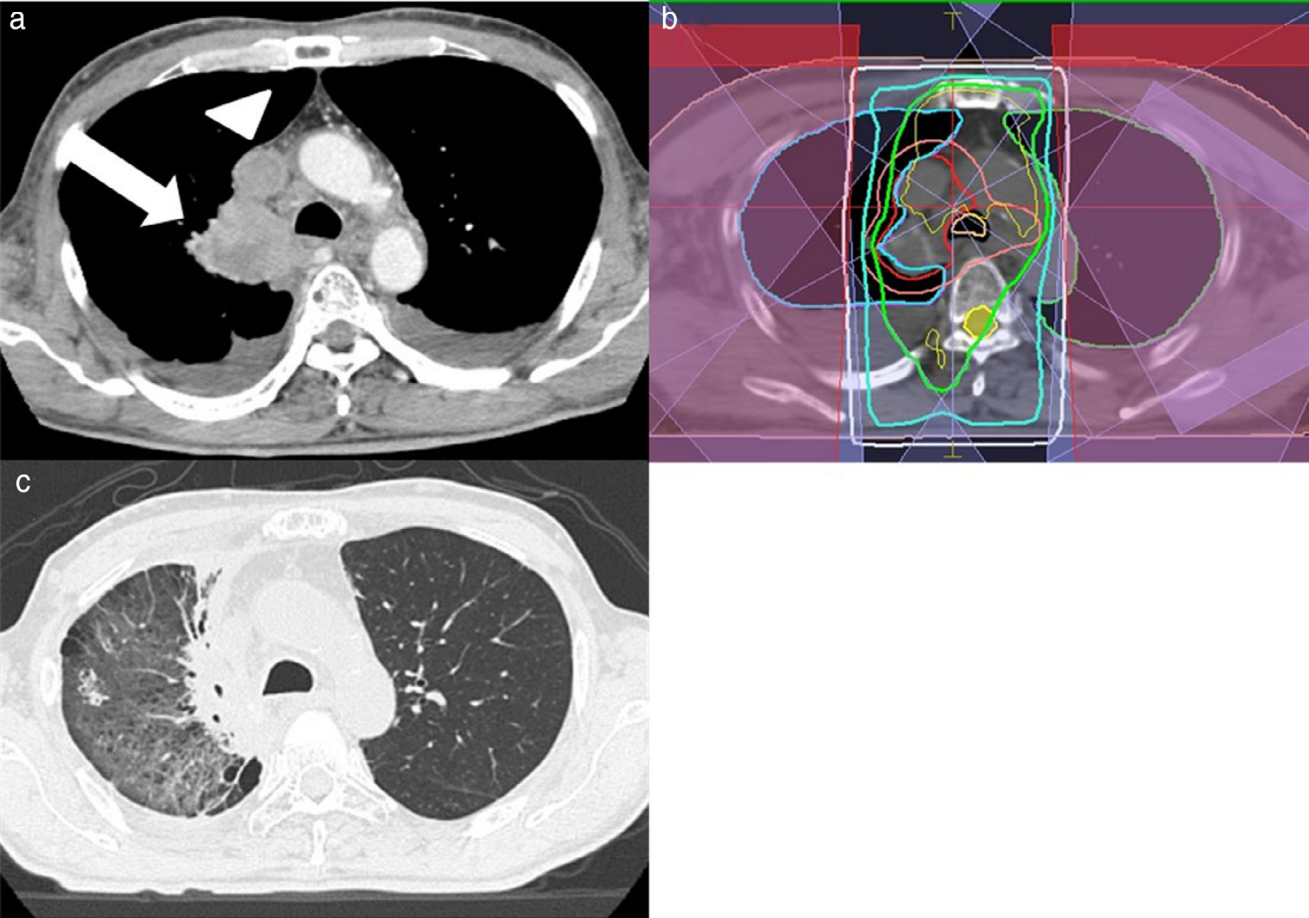
(**a**) Computed tomography (CT) shows primary lung cancer of the right hilum (arrow) with superior vena cava syndrome (arrowhead) in a man in his 60s. (**b**) He received three‐dimensional radiotherapy; isodose lines of 100% (yellow), 95% (light green), 85% (light blue), 50% (white), and the planned target volume (pink) are shown. (**c**) After 12 courses of an anti‐PD‐1 antibody (24 months after thoracic radiation therapy), CT showed new ground‐glass opacity in the irradiated area, which had also spread to the low‐dose area and outside the irradiated area. He was diagnosed with organizing pneumonitis.

The third case was of a man in his 60s who had an unknown pathological type of cancer in the right hilum (Fig 3a). He was diagnosed with cT4N2M0 stage IIIB NSCLC. He was administered three courses of neoadjuvant chemotherapy to reduce the radiation field and then received 3D‐CRT at a total dose of 60 Gy in 30 fractions (Fig 3b). Fourteen months after 3D‐CRT was completed, right pleural effusion had increased and tumor regrowth was observed. Thus, nivolumab was administered at 3 mg/kg (153 mg). Twelve months after the first nivolumab administration (27 months after 3D‐CRT), a diagnosis of grade 2 OP was made (Fig 3c).^7^

## Discussion

Radiation‐induced pneumonitis can be divided into two types: radiation pneumonia (RP) and OP.^8^, ^9^ While RP is direct damage as a result of radiotherapy, OP occurs via an autoimmune mechanism.^10^ Tsujino *et al.* reported that seven of 43 patients with small cell lung cancer administered chemoradiotherapy developed grade 2 or 3 pneumonitis.^11^ The incidence of any grade pneumonitis associated with PD‐1 inhibitor monotherapy in NSCLC patients has been reported as 4.1% (1.4–8.5%).^6^ Thus, patients administered nivolumab after TRT in our institute presented a higher OP rate than those administered anti‐PD‐1 antibody monotherapy or conventionally reported RP and OP, because of chemoradiotherapy. Tsujino *et al.* reported the mean V20 of RP grade 2 and 3 was 28.4% and 32.0%, respectively.^11^ In our report, V20 was considerably lower than that reported in previous studies^11^ however, OP occurred. OP after TRT has been attributed to an immunologically mediated mechanism, that is, an increase in the CD4/CD8 ratio detected on bronchioalveolar lavage.^12^ OP development may involve T cell and Fas/Fas‐ligand pathways, which can be activated by TRT.^13^, ^14^ In contrast, anti‐PD‐1 antibodies inhibit the PD‐1 pathway and increase the baseline T cell‐specific immune response, which in turn activates the cytotoxic immune response.^15^ However, dysfunction of these immune checkpoint molecules can lead to an imbalance in immunological mechanisms, which may result in irAEs; therefore, irAEs are thought to be principally T‐cell mediated.^16^ The mechanism and association between the two treatments remains unclear, possibly because of the synergistic adverse effects induced by TRT and nivolumab administration. The median time of pneumonitis appearance after anti‐PD‐1 antibody administration has previously been reported as approximately one month in patients with NSCLC.^17^, ^18^ Additionally, the time interval from TRT for lung cancer to OP development ranges from three to 18 months.^19^, ^20^ Thus, the influence of TRT may persist even when an anti‐PD‐1 antibody is administered some time after TRT is initiated.

These case reports draw attention to OP after TRT and anti‐PD‐1 antibody administration despite low V20. Careful follow‐up of such patients is advised considering synergistic adverse events.

## Disclosure

No authors report any conflict of interest.
